# Green Light Exposure Reduces Primary Hyperalgesia and Proinflammatory Cytokines in a Rodent Model of Knee Osteoarthritis: Shedding Light on Sex Differences

**DOI:** 10.3390/biomedicines12092005

**Published:** 2024-09-03

**Authors:** Laura Ventura, Renan F. do Espírito-Santo, Michael Keaser, Youping Zhang, Jin Y. Ro, Joyce T. Da Silva

**Affiliations:** 1 Center to Advance Chronic Pain Research, Department of Neural and Pain Sciences, University of Maryland Baltimore School of Dentistry, Baltimore, MD 21201, USAjro@umaryland.edu (J.Y.R.); jteixeira@umaryland.edu (J.T.D.S.); 2 Program in Neuroscience, University of Maryland Baltimore School of Medicine, Baltimore, MD 21201, USA

**Keywords:** phototherapy, chronic pain, inflammation, sex differences

## Abstract

Knee osteoarthritis (OA) often causes chronic pain that disproportionately affects females. Proinflammatory cytokines TNF-α, IL-1β, and IL-6 are key effectors of OA pathological changes. Green light shows potential as an alternative intervention for various pain conditions. However, no studies have investigated green light′s analgesic effects in both sexes in chronic knee OA. We induced unilateral knee OA with intra-articular injection of monoiodoacetate (MIA) in male and female Sprague-Dawley rats. Two days post-injection, the rats were exposed to green-light-emitting diodes (GLED) or ambient room light eight hours daily for 24 days. Knee mechanical sensitivity was assessed using a small animal algometer. Blood serum concentrations of TNF-α, IL-1β, IL-6, and IL-10 were quantified at baseline and 23 days post-injection. MIA injection decreased the knee mechanical thresholds of the male and female rats. GLED exposure attenuated mechanical hypersensitivity in both sexes compared to the controls; however, GLED-induced analgesia occurred sooner and with greater magnitude in males than in females. In both sexes, the analgesic effects of green light lasted 5 days after the final GLED session. Finally, GLED exposure reversed the elevation of serum proinflammatory cytokines. These findings suggest that GLED exposure reduces primary hyperalgesia in OA, potentially by lowering proinflammatory cytokines, and indicate sex differences in GLED-induced analgesia.

## 1. Introduction

Based on National Health Interview Survey data spanning 2019–2021, the Center for Disease Control and Prevention estimated a chronic pain prevalence of 20.9% among US adults (51.7 million), with females and older adults overrepresented in this number [1]. When stratified by chronic medical condition, the prevalence of chronic pain within the arthritic population was among the highest. The most common form of arthritis, osteoarthritis (OA), remains a leading cause of musculoskeletal-related disability worldwide [2], and a substantial patient population presents with persistent pain refractory to standard pharmacological and non-pharmacological interventions [3,4]. Although increasing age has been consistently recognized as a predominant risk factor for developing OA, factors besides age, such as female sex, previous joint injury, physically demanding occupations, and obesity, have also been associated with the development of OA [5,6,7]. The extent of the burden of OA is demonstrated in its profound impact on other organ systems, engendering metabolic, cardiovascular, neurological, and mental health complications [8], which may disproportionately affect female patients [9]. These comorbidities, present in nearly half of OA patients, typically exacerbate OA and complicate treatment approaches; for example, the use of NSAIDs, a common treatment for OA-related pain, may increase the risk of adverse side effects in this vulnerable population [10]. The global rise in OA cases [11] deepens the need for pain management approaches that demonstrate long-term efficacy and safety, as current therapeutics still provide inadequate pain relief and often cause undesirable effects [4,12,13,14,15].

Although OA necessarily entails the loss of cartilage, it is now better defined as a chronic disease of the whole joint that can be characterized not only by cartilage degeneration, but also by remodeling of subchondral bone, joint inflammation, and the weakening of surrounding muscle/ligaments [16,17]. These features are classically accompanied by pain, stiffness, and limited mobility in the affected joint(s) [18]. More specifically, in knee OA, the key effectors of these pathological changes are the proinflammatory cytokines tumor necrosis factor α (TNF-α), interleukin 1β (IL-1β), and IL-6 [19,20]. The alteration of the cytokine milieu towards greater concentrations of proinflammatory cytokines may be a primary mechanism in the initiation and maintenance of OA [21,22,23,24,25]. Elevated serum and synovial fluid levels of TNF-α, IL-1β, and IL-6 have been positively correlated with pain severity in early- and late-stage knee OA [26,27,28,29,30,31,32]. However, it should be noted that there has been contradictory evidence concerning serum and synovial fluid cytokine levels and pain severity in human OA patients, possibly due to factors such as OA stage, patient age, and type of pain assessment.

Several animal models have been developed in an effort to elucidate the mechanisms driving OA-related pain, including spontaneous and surgically or chemically induced models [33,34]. One chemically induced OA model leverages the aerobic glycolysis inhibitor monoiodoacetate (MIA) to disrupt chondrocyte activity. This results in the rapid development of histological and pain-like phenotypes similar to those seen in human OA, thereby facilitating the investigation of OA-related pain in a relatively short period [35,36,37,38]. Knee tissue levels of TNF-α and IL-6 have been shown to increase one day after a single intra-articular knee injection of MIA (2 mg/25 μL), peaking four days and remaining elevated 28 days after injection [39]. Serum levels of TNF-α, IL-1β, and IL-6 are elevated in both early and late stages of the MIA model, mirroring findings in OA patients and suggesting their use as diagnostic biomarkers for predicting OA risk and progression [40,41]. 

Current interventions for knee OA largely focus on pain management and include pharmacological and non-pharmacological approaches, such as oral and topical NSAIDs, intra-articular corticosteroid injections, exercise, and knee bracing [42]; however, these approaches are limited by (1) adverse side effects and limited efficacy, (2) cost and other barriers to accessibility, and (3) the exacerbation of pain with exercise [4,12,13,14,15,43,44]. Safe, accessible treatments that overcome these barriers are sorely needed. Light therapy has emerged as a potential cost-effective and low-risk intervention for various pain conditions [45]. Green-light-emitting diode (GLED) exposure, in particular, has been shown to elicit long-lasting analgesic effects in clinical [46,47] and preclinical studies [48,49,50,51,52,53]. In a model of post-surgical pain, GLED exposure increased cerebrospinal fluid (CSF) levels of IL-10 and decreased TNF-α, which was consistent with reduced microglia activation in the spinal cord [51], showing that GLED exposure attenuates inflammatory processes. However, to the best of our knowledge, no studies have examined the effects of green light on prolonged inflammatory pain in the joint, especially considering sex as a determinant for analgesia. 

Here, we interrogated the effects of GLED exposure on knee mechanical hypersensitivity (primary hyperalgesia) and blood cytokine levels in the MIA model of knee OA using male and female Sprague-Dawley rats. Sex differences in systemic cytokine levels in patients with painful knee OA have been reported [54], and preclinical studies have shown that the mechanisms underlying pain and inflammatory processes are modulated by sex [55,56,57,58,59]. However, less is known about how sex may mediate the effects of non-pharmacological pain interventions, such as green light therapy. Thus, we evaluated potential sex differences in the effects of GLED exposure on MIA-induced knee pain and serum cytokine levels, with the overarching hypothesis that GLED reduces OA-like knee pain through its anti-inflammatory effects in a sex-dependent manner. Here, we report that a single intra-articular injection of MIA significantly decreased knee mechanical thresholds of male and female rats. GLED exposure reversed primary hyperalgesia in both male and female rats compared to ambient room light (ARL) controls, with the analgesic effects of GLED exposure occurring sooner and with greater magnitude in the males. Blood serum samples collected at baseline and 23 days after the MIA injection revealed an anti-inflammatory effect of the GLED exposure, suggesting that GLED′s analgesic effects in the MIA model may be partly mediated by an attenuation of circulating proinflammatory cytokines. The findings presented herein add to the evidence supporting green light analgesia in various chronic pain conditions, specifically demonstrating its potential as an accessible and low-risk intervention for patients with knee osteoarthritis, who commonly experience inadequate pain relief with standard treatment approaches. Moreover, this work presents novel findings regarding sex-based differences in the treatment effectiveness of green light exposure for primary hyperalgesia. Overall, this work will facilitate future investigations into the mechanisms of green light analgesia, with the goal of adapting and optimizing this intervention for patient use. 

## 2. Material and Methods

### 2.1. Animals 

Adult male and female Sprague-Dawley rats (weight at testing 340–360 g and 213–224 g, respectively; 3–5 months old) were used for this study. All animals were housed in a climate-controlled room under a 12 h light–dark cycle with access to food and water ad libitum. All procedures were performed in accordance with the National Institutes of Health Guide for the Care and Use of Laboratory Animals and under a University of Maryland–approved Institutional Animal Care and Use Committee protocol. Rats were allowed a minimum of 7 days of acclimation in the housing facility before being acclimated to experimenters for one week prior to experimentation.

### 2.2. Unilateral Monoiodoacetate (MIA) Knee Injection 

To induce knee osteoarthritis, a single injection of MIA was administered to the left knee joint. Rats were anaesthetized with isoflurane in O_2_ at an induction dose of >3–4.5% followed by a maintenance dose of >1.5–3% for the duration of the procedure. Fur was clipped from the surface of the left knee, and the site of injection was cleansed with 70% isopropyl alcohol, followed by 7.5% povidone-iodine. A total of 3 mg of MIA in 15 µL of 0.9% saline was injected into the left knee joint through the infra-patellar ligament using a 27-gauge needle. 

### 2.3. GLED Exposure 

Two days after MIA injection, rats were exposed to green or ambient room light as a control 8 h daily (7:30 a.m. to 3:30 p.m.) during the light phase for 24 days (adapted from [48]). Green-light-emitting diode (GLED) strips (#LS-AC50-GR-6, 520 nm in wavelength, 13.8 W, 120 V, 120° beam angle) were purchased from ledsupply.com (LEDSupply, Randolph, VT, USA). As the control condition, ambient room light (ARL) was provided by regular fluorescent bulbs, consistent with lighting conditions in the animal holding facility. For the daily light exposure sessions, animals were transported to separate, climate-controlled rooms and transferred from their semi-transparent home cages to fully transparent cages with lids that allow light exposure from above. To achieve illumination with green light at an intensity between 10 and 100 lux, two GLED strips were affixed to the horizontal bar of a 5-foot-by-8-foot rectangular PVC stand so that the GLED strips were orientated parallel to the cages. The GLED strips were connected to an outlet timer to facilitate automated control over the start and stop times of green light exposure. To ensure uninterrupted green light exposure, a remotely accessible webcam was positioned in the room, and the environment was kept devoid of any light sources other than the GLED strips. In a separate room, the control group was exposed to ARL (10 to 30 lux), as detailed above. The light intensity inside ARL and GLED cages was measured in lux using a lux meter, which provides a reading of illuminance in a given area but does not directly correspond to its radiometric counterpart, irradiance, at specific wavelengths. The low-level lux intensity of the ARL room was specifically chosen to match typical lighting conditions in the animal holding facility while also reducing irradiance relative to GLED room lighting. Behavioral assays were performed immediately after light exposure termination at 3:30 p.m. After testing, animals were promptly returned to their home cages and transported back to the holding facility.

### 2.4. Knee Mechanical Threshold 

Mechanical sensitivity was tested at the left knee joint using a small animal algometer (SMALGO, Bioseb, Vitrolles, France). Experimenters gently covered the torso of the rat with a towel to minimize distress during testing. Pressure was applied to the lateral joint line of the left knee with the tip of the SMALGO stimulator unit until the rat withdrew its leg. Two measurements were averaged for the left knee of each rat at each time point (baseline, one day after MIA injection, every two days up to 24 days after MIA injection, and five days after treatment end—30 days after MIA injection). To standardize the rate of pressure application, SMALGO was monitored in real time with force curves displayed through the BIO-CIS2 software version 1.5.1.0, which can be connected to the Bioseb instrument control unit. Pressure was increased progressively according to the parameters set on the BIO-CIS2 software: a maximum threshold of 1500 g and a test duration of 2 s, which represents a constant rate of 187.5 g/250 ms. These parameters were the same for each animal on each testing day. The force, reported in grams, which elicited a withdrawal response was recorded as the mechanical threshold. Rats were acclimated to the experimenter, testing room, and restraint towel for one week prior to experimentation. 

### 2.5. Blood Serum Collection and Cytokine Quantification

Blood samples were obtained from the ventral tail artery to assess levels of key cytokines TNF-α, IL-1β, IL-6, and IL-10. Rats were anaesthetized with isoflurane in O_2_ (induction: >3–4.5%; maintenance: >1.5–3%) for all blood collection procedures. Blood samples were centrifuged to separate the serum and stored at −20 °C. Blood samples were collected at 2 p.m. at each blood-collection time point (baseline and after 22 sessions of GLED or ARL, which was 23 days post-MIA injection). After blood collection, animals were promptly returned to their home cages and transported back to the holding facility. Samples were sent to the University of Maryland School of Medicine Cytokine Core Laboratory to measure the concentration of cytokines (pg/mL) in the serum samples using a Luminex™ 100 multi-analyte system with their exponent software version 4.3 (Luminex Corporation, Austin, TX, USA).

### 2.6. Statistical Analysis

Results were analyzed using the statistical analysis software, GraphPad Prism version 10.2.2. Mixed-effects model with Tukey′s correction of multiple comparisons was performed to determine significant (1) treatment and time effects on mechanical thresholds (raw data in g) for each sex group, males and females; (2) sex and time effects on mechanical thresholds for each treatment group, GLED and ARL (data transformed to percentage and normalized to each group′s baseline average, since groups showed differences in baseline mechanical sensitivity due to specific sex); (3) sex effect on the percentage difference in mechanical threshold of GLED-exposed male and female rats relative to their sex-matched ARL counterparts; and (4) sex and time effects on the cytokine data. Two-way repeated measures ANOVA with uncorrected Fisher′s LSD was performed to determine significant treatment and time effects on the cytokine data for each sex group, males and females. Differences were considered statistically significant at *p* < 0.05 and the data were presented as individual values or mean ± standard deviation (SD).

## 3. Results

### 3.1. Green Light Reduces Primary Hyperalgesia in a Model of Chronic Knee Osteoarthritis in Males and Females, with Effects Lasting beyond Therapy Termination

**Figure 1:**
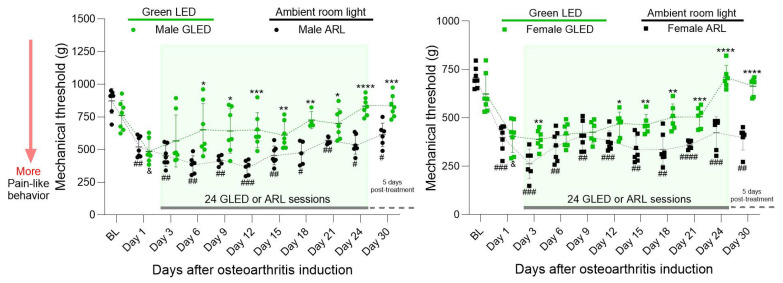
GLED exposure attenuates mechanical hypersensitivity in male (**left**) and female (**right**) rats in the MIA model of osteoarthritis. Mechanical thresholds of the knee were assessed before and after MIA injection. The male rats exposed to GLED exhibited a significant reduction in mechanical hypersensitivity after five sessions (Day 6), while the females showed a significant reduction at the 11th GLED session (Day 12), compared to their ARL counterparts. The GLED′s analgesic effects were maintained 5 days after therapy termination. Group data were staggered for readability; all the groups were tested at the same time points. Each point represents data from one animal. The horizontal bars show the mean and the error bars show ± SD. n = 6–7 (* *p* < 0.05, ** *p* < 0.005, *** *p* < 0.0005, **** *p* < 0.0001 for group differences; # *p* < 0.05, ## *p* < 0.005, ### *p* < 0.0005, #### *p* < 0.0001 BL versus time points in ARL group, & *p* < 0.05 BL vs. time points in GLED group). BL = baseline.

To determine the effects of GLED exposure on MIA-induced hyperalgesia, mechanical thresholds were measured by progressively applying pressure to the lateral joint line of the left knee until withdrawal of the hind leg. In male rats, mechanical thresholds significantly decreased one day after MIA injection compared to baseline (& *p* < 0.05 for GLED group and ## *p* < 0.005 for ARL group), indicating the development of primary hyperalgesia (Figure 1). The mechanical thresholds of GLED-exposed males were not significantly different from baseline three days post-MIA injection (day 3, two GLED sessions) or for the remaining time points. GLED exposure significantly increased mechanical thresholds relative to the ARL condition after five GLED sessions (six days post-MIA injection; * *p* < 0.05). The mechanical thresholds of GLED-exposed males remained significantly different from male ARL mechanical thresholds at all remaining time points, including five days after GLED exposure termination, supporting the long-lasting analgesic effects of GLED exposure reported in previous studies (* *p* < 0.05, ** *p* < 0.005, *** *p* < 0.0005 and **** *p* < 0.0001) [48,49,50,52,53]. Conversely, although the mechanical thresholds of ARL-exposed males increased slightly from day 12, they remained significantly different from baseline at all time points, suggesting that these animals never completely recovered from MIA-induced knee hyperalgesia (# *p* < 0.05, ## *p* < 0.005, ### *p* < 0.0005 and #### *p* < 0.0001). The mechanical thresholds of the female rats significantly decreased one day after MIA injection compared to baseline (& *p* < 0.05 for GLED group and ### *p* < 0.0005 for ARL group) (Figure 1). Similarly to their male counterparts, the mechanical thresholds of GLED-exposed females were not significantly different from baseline three days post-MIA injection or at subsequent time points. Although a significant difference from ARL-exposed females emerged at this time point (day 3; ** *p* < 0.005), the effect was not maintained. Rather, GLED exposure significantly and stably increased mechanical thresholds relative to the ARL condition at the 11th GLED session (12 days post-MIA injection; * *p* < 0.05), remaining significantly different at all remaining time points (** *p* < 0.005, *** *p* < 0.0005, and **** *p* < 0.0001). The analgesic effects of green light were maintained for an additional five days after GLED exposure termination (**** *p* < 0.0001).

### 3.2. Sex Differences in Primary Hyperalgesia in a Model of Chronic Knee Osteoarthritis 

**Figure 2:**
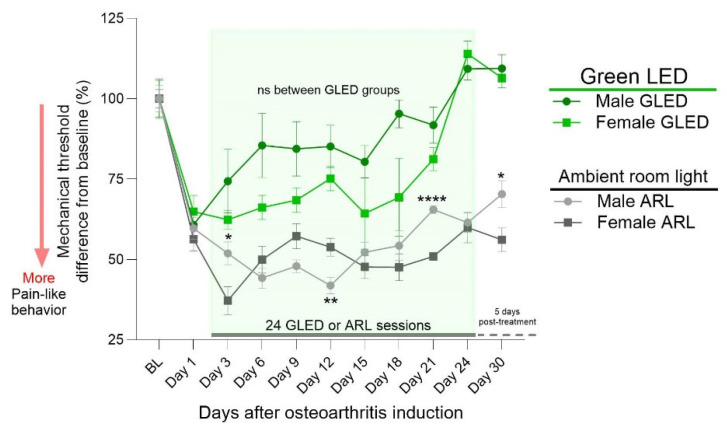
The percentage change in mechanical threshold from baseline revealed a sparse sex difference in knee hypersensitivity between the ARL groups, but not the GLED groups. The male and female rats across both groups showed comparable drops in mechanical threshold 1 day after MIA injection. However, at days 3, 12, 21, and 30, the male and female rats exposed to ARL showed a significant difference in mechanical threshold. Although it did not reach statistical significance, the GLED-exposed females showed a trend of a greater decrease in mechanical threshold from baseline relative to their male counterparts. The data are mean with ± SD. n = 6–7. (* *p* < 0.05, ** *p* < 0.005, **** *p* < 0.0001 male ARL vs. female ARL). BL = baseline.

To assess the effect of sex on mechanical thresholds within the treatment-matched groups over time, the raw data in grams were normalized to group-specific baselines and represented as percentage changes from baseline (Figure 2). With this approach, the differences in baseline mechanical threshold could be accounted for (Figure 1). Significant differences throughout the ARL sessions emerged at day 3, day 12, day 21, and day 30 (* *p* < 0.05, ** *p* < 0.005 and **** *p* < 0.0001 male ARL vs. female ARL groups), confirming potential sex differences within the MIA model, as previously reported [60,61]. Significant differences did not emerge at any of the time points in the GLED-exposed groups. 

### 3.3. Magnitude of GLED Effects on Males and Females Relative to Their Sex-Matched Controls

**Figure 3:**
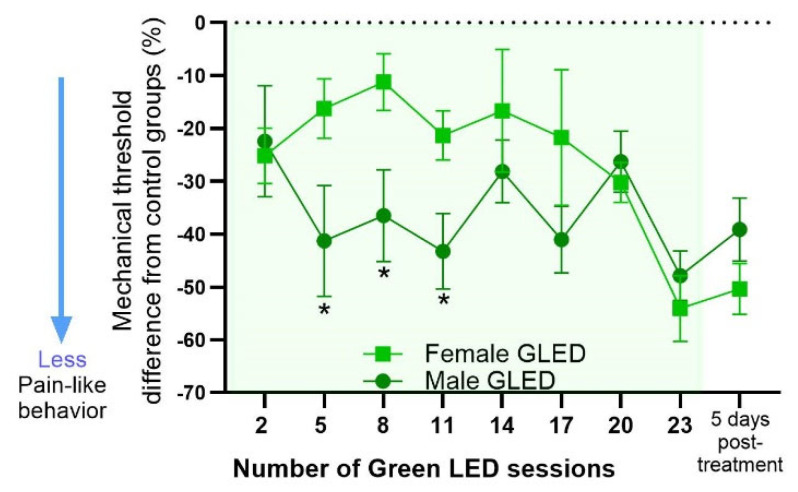
The percent difference in mechanical thresholds of GLED-exposed male and female rats relative to their sex-matched ARL counterparts revealed that male rats exhibited greater treatment effects than females. At GLED sessions 5, 8, and 11, male rats showed significant differences in mechanical threshold (%) compared to female rats. Data are mean with ± SD. n = 6–7 (* *p* < 0.05).

The normalized data (Figure 2) were further analyzed to test whether the magnitude of GLED′s analgesic effects differed between males and females by comparing the difference in mechanical threshold between GLED-exposed male and female rats relative to their sex-matched ARL counterparts. Significant differences between GLED-exposed males and females were observed from the fifth to the eleventh GLED session, suggesting a differential timing of the GLED′s analgesic effects based on sex (Figure 3; * *p* < 0.05). 

### 3.4. Green Light Reduces Serum Proinflammatory Cytokine Levels 

**Figure 4:**
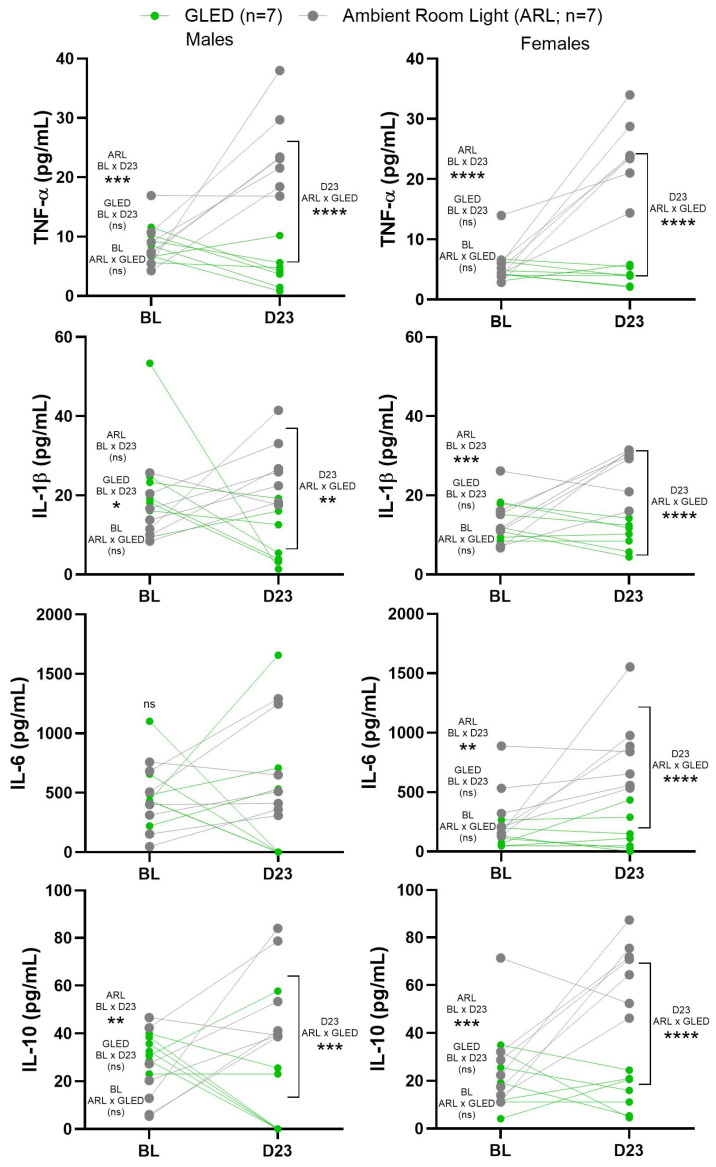
Blood serum cytokine levels at baseline and after 22 sessions of GLED or ARL (23 days after MIA injection). Male and female rats exposed to GLED showed significantly lower levels of cytokines TNF-α, IL-1β, and IL-10 at D23 compared to their ARL counterparts. Female rats, but not male rats, exposed to GLED showed significantly lower levels of IL-6 compared to their ARL counterparts at D23. ELISA assay results representing the average TNF-α, IL-1β, IL-6, and IL-10 concentrations in the serum samples at baseline and 23 days after osteoarthritis induction. All the results represent individual values, with the p values obtained from statistical analyses (* *p* < 0.05, ** *p* < 0.005, *** *p* < 0.0005, **** *p* < 0.0001). ns indicates not significant. n = 7. BL = baseline, D23 = Day 23.

**Figure 5:**
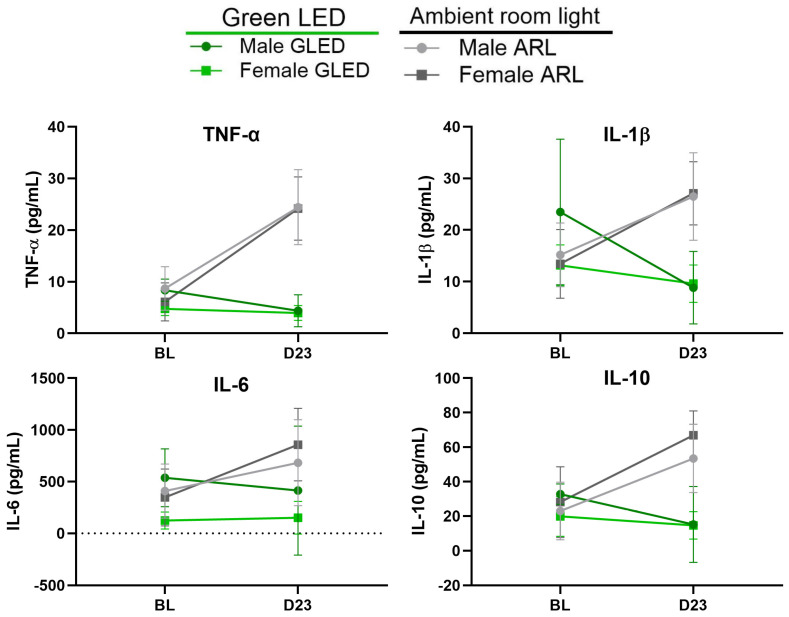
No sex differences in blood serum cytokine levels at baseline or after 22 sessions of GLED or ARL (23 days after MIA injection). Data are mean with ± SD. n = 7. BL = baseline, D23 = Day 23.

To assess the anti-inflammatory effects of GLED exposure in the MIA model, blood serum samples were collected at baseline and 23 days post-MIA injection (D23; 22 GLED sessions) and evaluated for the concentrations of TNF-α, IL-1β, IL-6, and IL-10. The baseline levels of all the cytokines were similar between all groups (Figure 4 and Figure 5). A sex-specific analysis showed that the serum levels of TNF-α increased from baseline to D23 in both ARL-exposed male and female rats (*** *p* < 0.0005 for males and **** *p* < 0.0001 for females), while they remained unchanged for the GLED-exposed groups. TNF-α levels were significantly higher at D23 in the ARL condition compared to the GLED groups for both sexes (Figure 4; **** *p* < 0.0001). In ARL-exposed males, serum levels of IL-1β did not change from baseline. GLED-exposed males showed a decrease relative to their baseline measure (* *p* < 0.05) and the control group, with GLED D23 serum levels of IL-1β being significantly lower than those of the ARL-exposed males (Figure 4; ** *p* < 0.005). IL-1β increased from baseline in ARL-exposed females (*** *p* < 0.0005), while it remained unchanged in GLED-exposed females, with a significant difference at D23 between ARL and GLED groups (**** *p* < 0.0001). No differences appeared in the serum levels of IL-6 in males from any group (Figure 4). In ARL-exposed females, IL-6 was increased at D23 (** *p* < 0.005) but remained at baseline level in GLED-exposed females at this time point. Surprisingly, IL-10, a prototypical anti-inflammatory cytokine, increased from baseline in both ARL-exposed male and female rats (** *p* < 0.005 for males and *** *p* < 0.0005 for females), while its levels remained unchanged for the GLED-exposed counterparts (Figure 4), with significant differences at D23 between ARL and GLED groups (*** *p* < 0.0005 for males and **** *p* < 0.0001 for females). No significant differences at baseline or D23 were observed between sexes in the treatment-matched groups (Figure 5). 

## 4. Discussion

Our results provide evidence for the analgesic effects of GLED therapy in the MIA-induced OA pain model. Daily GLED exposure attenuated primary hyperalgesia in the knee, significantly increasing mechanical thresholds during the early and late stages of the MIA model over the course of the 24 light-exposure sessions, with these changes occurring sooner in male rats. In addition, the attenuation of mechanical hypersensitivity was accompanied by a reduction in circulating proinflammatory cytokines. Consistent with previous studies, GLED’s analgesic effects exceeded the duration of the light exposure (5 days). These data add to the growing body of research demonstrating GLED′s analgesic effects in various chronic pain conditions and highlight its potential as a cost-effective, low-risk therapy for patients with painful knee osteoarthritis. 

Previous studies investigating green light analgesia generally exposed rodents to green light for 4–7 days, depending on the pain model, with the reversal of hypersensitivity occurring within this timeframe [48,49,50,52]. Based on the protocol proposed by Ibrahim et. al. [48], here, we initially exposed rats to GLED for 8 h daily for 5 days and measured the mechanical sensitivity at the knee every two days. The within-group comparisons showed no differences from baseline after two GLED sessions. However, the group comparisons showed that while 5 days of GLED exposure (6 days post-injection) significantly increased the mechanical thresholds of GLED-exposed males compared to their ARL counterparts, this exposure duration was insufficient for females. We found that whereas fewer GLED sessions elicited analgesia in males, green light started to reduce mechanical hypersensitivity significantly in females by the 11th GLED session (12 days post-injection) compared to ARL-exposed females. When the GLED groups were directly compared for sex differences based on the mean difference from their ARL counterparts, this analysis confirmed that the analgesic effects of the green light exposure occurred sooner in males. MIA induces long-lasting inflammatory pain at the site of injection, with female rats exhibiting a longer recovery time than their male counterparts in the MIA-induced knee OA model [60]. Although GLED′s analgesic effects generally occurred earlier in the pain models examined in previous studies, the nature of the MIA model, which comprises early and late stages of disease progression and pain-like behaviors [62,63] combined with a measure of mechanical hypersensitivity specific to the site of injury (primary hyperalgesia), may explain the slower onset of green light analgesia, which was particularly observed in the females. 

Previous clinical and preclinical studies have consistently shown that white LED does not elicit analgesia across different pain conditions. Thus, we opted to use the existing fluorescent lighting available in our ambient room light (ARL) space as our control lighting condition to match the lighting conditions of the animal holding facility. Both groups underwent the same daily procedures, including (1) transportation to a room designated for light therapy and (2) additional handling due to the rats’ being moved to transparent cages for the light exposure. Analysis of the groups in the ARL condition confirmed that there are sex differences within the MIA model, which is consistent with our previous study comparing the effects of sex and age in this model [60]. Given that there are sex differences in the prevalence, severity, and treatment effectiveness of knee OA [64,65,66,67], the results reported herein, at the preclinical level, are in agreement with the existing evidence for the impact of sex in knee OA pain and its management. However, the mechanisms by which males benefit sooner from this light intervention are yet to be elucidated.

Our data demonstrate a lack of sex differences in knee-pain-like behavior and serum cytokine levels in the late, or chronic, OA stage in GLED-exposed animals, which does not support our initial hypothesis. Sex differences in cytokine levels were not observed at baseline or day 23 in the treatment-matched groups. Day 23 post-MIA injection represents a resolution in OA-like pain behavior for both GLED-exposed male and female rats, which exhibited similar responses relative to their baselines at this time point. This is consistent with the similarity in cytokine levels; thus, it is possible that an earlier time point, when sex differences in pain-like behavior are present, could reveal sex differences in cytokine levels. Other mechanisms underlying pain processing at multiple levels of the neuroaxis that do not directly involve inflammatory cytokines may also explain the sex differences in the GLED′s analgesic effects.

A recent study demonstrated sexual dimorphism at the nociceptor level across mice, rhesus monkeys, and humans, providing a mechanism for sex-based differences in pain starting from the periphery and showing that such differences are conserved across species [56]. In a model of post-surgical pain using both male and female rats, GLED reduced the expression of spinal Iba1 (a marker of microglial activation) in both sexes, but microglia from females exhibited more complex arborization and longer processes compared to males, suggesting that green light has central anti-inflammatory effects that may be differentially affected by sex [51]. Comparably, increased microglial activity in the spinal cord has been reported in the MIA-induced OA model in rats [68]. However, that study did not evaluate the effects of sex. 

Our previous work identified sex- and age-dependent differences in brain functional connectivity [69] and in pain-like behaviors in the MIA model, with males displaying less hyperalgesia [60]. Moreover, in our investigation of the descending pain modulatory system, healthy male rats exhibited stronger endogenous pain inhibitory responses than their female counterparts, which corresponded with greater connectivity between the anterior cingulate cortex (ACC) and the periaqueductal gray (PAG) [70]. We also observed that young male rats showed increased connectivity between ACC and PAG in the progression from early to late stage OA, which may explain the faster recovery in this group. Females, on the other hand, displayed more widespread PAG connectivity, including regions associated with pain and emotional processing. As green light analgesia has been shown to engage brain regions involved in the descending pain modulatory system, namely the ACC [53], rostral ventromedial medulla [48], and dorsal raphe nucleus [50], males may benefit sooner from GLED exposure through the potentiation of specific pain modulatory circuits that are postulated to facilitate pain control in a sex-dependent manner in the MIA model.

As elevated cytokine concentrations in the serum and synovial fluid have been correlated with the disease progression and pain symptoms of knee OA patients, we were interested in investigating the effects of GLED exposure on the serum levels of proinflammatory cytokines TNF-α, IL-1β, and IL-6, as well as a prototypical anti-inflammatory cytokine, IL-10. These proinflammatory cytokines have been shown to be elevated in serum and synovial fluid in the MIA model [71,72,73,74,75]. Our results show that, in the absence of a therapeutic intervention (ARL groups), serum cytokine levels were elevated in the later stage of the MIA model (day 23). However, GLED exposure may have reversed or prevented this proinflammatory process, as evidenced by the reductions in serum TNF-α and IL-1β in both males and females, and in IL-6 levels in females. Notably, the anti-inflammatory cytokine, IL-10, was also elevated in the ARL groups at day 23. This may reflect immune homeostasis, as IL-10 is known to respond to inflammatory stimuli, such as TNF-α, and dampen proinflammatory cytokine production [76]. The lack of IL-10 elevation in the GLED-exposed groups at day 23 may potentially be explained by GLED′s attenuation of circulating proinflammatory cytokines throughout the 22 sessions. 

Inflammatory processes within the knee joint have been shown to elicit systemic inflammation in OA via increased levels of proinflammatory cytokines in the serum, such as IL-1β [77]. The ARL groups exhibited elevated serum proinflammatory cytokine levels, including those of IL-1β, TNF-α, and IL-6. This pathological change was reversed by GLED exposure, suggesting that this light intervention may induce systemic anti-inflammatory effects. The potential mechanisms underlying these effects may include both direct and indirect neuroimmune interactions. In OA patients, a correlation between CSF and serum levels of proinflammatory markers has been reported, including those that increase the permeability of the blood-brain barrier [78]. Persistent peripheral inflammation can increase the transfer of proinflammatory cytokines from the periphery to the CNS across the blood-brain barrier [78]. Considering that green light has been shown to modulate neuroinflammatory processes [51], these findings suggest a possible overlap or bidirectional crosstalk between central and peripheral anti-inflammatory signaling. GLED exposure could act either directly (e.g., through neuroimmune interactions involving nociceptors) or indirectly (e.g., by modulating the stress axis and neurocircuitries related to pain, sleep, and mood) to modulate cytokine signaling. To address the limitations of the current study, further research is warranted to mechanistically elucidate how green light may alter serum cytokine levels. This should include assessments in the early stages of the MIA model and an investigation into how longitudinal changes in cytokine levels may differ between sexes.

## 5. Conclusions

The results reported herein indicate that green light exposure attenuates primary hyperalgesia in the MIA model of knee OA, with a faster onset of analgesia in males compared to females. Given that knee OA-related pain in patients and rodent models is accompanied by the elevation of serum proinflammatory cytokines, GLED′s analgesic effects in the rat MIA model may be due, at least in part, to the reduction in circulating proinflammatory cytokines, such as TNF-α, IL-1β, and IL-6. While the mechanisms underlying males experiencing benefits sooner from GLED exposure are yet to be elucidated, the current findings highlight that treatment strategies involving green light may need to be tailored for men and women. 

## Data Availability

Data are available upon reasonable request.
